# Past, present and imaginary: Pathography in all its forms

**DOI:** 10.1177/13634593211060759

**Published:** 2021-11-25

**Authors:** Annemarie Jutel, Ginny Russell

**Affiliations:** Te Herenga Waka | Victoria University of Wellington, New Zealand; University of Exeter, UK

**Keywords:** autism spectrum disorder, diagnosis, pathography

## Abstract

Diagnosis is a profoundly social phenomenon which, while putatively identifying disease entities, also provides insights into how societies understand and explain health, illness and deviance. In this paper, we explore how diagnosis becomes part of popular culture through its use in many non-clinical settings. From historical diagnosis of long-deceased public personalities to media diagnoses of prominent politicians and even diagnostic analysis of fictitious characters, the diagnosis does meaningful social work, explaining diversity and legitimising deviance in the popular imagination. We discuss a range of diagnostic approaches from paleopathography to fictopathography, which all take place outside of the clinic. Through pathography, diagnosis creeps into widespread and everyday domains it has not occupied previously, performing medicalisation through popularisation. We describe how these pathographies capture, not the disorders of historical or fictitious figures, rather, the anxieties of a contemporary society, eager to explain deviance in ways that helps to make sense of the world, past, present and imaginary.

Medical sociologists have been key in drawing attention to diagnosis as a focus for research and scholarship (Blaxter, 1978; Brown, 1995). Within a sociological framework, diagnosis is not only a method of classification of disease, but a time-bound process that involves many players and much negotiation (Jutel and Nettleton, 2011). Studies have explored the task of diagnosis, not just as a process of categorising, but also as an interpretation of bodily signs and mental distress as symptoms, with some scholars focussing specifically on diagnostic tests and how these interact with medical and patient authority, the formulation and negotiation of diagnosis, (Brossard and Carpentier, 2017; Ebeling, 2011; Jutel, 2013). Others have explored the consequences of diagnosis, and how diagnosis is not a neutral process of discovery of disease, but itself impacts health, thus is itself a form of intervention (Russell et al., 2012). Diagnosis has consequences in its own right, both in terms of prognosis, treatment pathways and health, but by assigning one or receiving and accepting one, there are often subtle changes in the way diseases are enacted and understood, and these feed back into the understandings and definitions of the categories themselves (Hacking, 1996). Diagnosis, then, is an altogether complex business.

But diagnosis is also used outside of the clinical encounter on real and on imaginary characters. This could be the diagnoses of historical figures, or of fictional ones: sometimes by a medical pundit, other times by a social commentator, a member of the public or a novelist. We see these extra-clinical diagnoses every day, in newspaper reports, movies and other aspects of popular culture. These are diagnoses which are not clinical and may not be contemporaneous, but still have the capacity to story a life.

In this paper, we utilise the term ‘pathography’ to describe these extra-clinical diagnoses in order to encapsulate their multiple and overlapping tasks; and analyse who is diagnosing whom-and why. These pathographies play an important role in contemporary culture, providing shape to the stories we tell, and also shining a light on popular expectations of diagnosis.

In the clinical setting, diagnosis occurs after an individual seeks an explanation for an ailment. The usual process is for a patient to approach the diagnostician with a story about symptoms and feelings which they see to be associated with disease. They hope to be given a diagnostic label to explain their illness, because it imposes order on the chaos disorder of disease, and it brings with it a treatment and a plan.

Today, more than ever before, diagnostic language has crept into everyday talk. There is a popular fascination with diagnosis and diagnostic language beyond the clinic, for the explanatory potential that they can offer. With a word or a phrase, we can paint a picture, tell a story. There is no need to explain symptoms, because they are captured in the diagnostic label. Instead of having to detail symptoms and causative agent, one word can explain the case. And as in the case of clinical diagnosis, in these extra-clinical stories, the social roles of diagnosis are implemented in the service of history, fiction, politics and so forth. The diagnosis legitimises (Parsons, 1958) or alternatively stigmatises (Scambler, 2009) and explains deviance (Conrad and Schneider, 1992), but not always in the service of the sick role, rather, in the service of story-telling.

The seepage from the clinic of diagnostic language has been facilitated by mass communication on-line and in digital platforms where exploring medical symptoms and concepts – ‘doing diagnosis’ – has become a popular past-time and accepted form of entertainment. Popular culture (e.g. blockbuster TV shows such as *House*, use diagnostic mysteries to underpin plots, less *whodunit* and more *whatsit*). Meanwhile outside the clinic, individuals are all experts in internet research and are increasingly encouraged to take responsibility for identifying their own ailments. Diagnosis has become a mediator of medicalisation, seeping into everyday domains previously left to clinical expertise.

In western, high-income societies, which form the context of our discussion, diagnosis is used by multiple interacting institutions, so woven into the fabric of our institutions that it is impossible to imagine life without it (Rosenberg, 2002). Increasingly, western concepts of health illness and diagnosis are exported elsewhere and often adapted. This ‘diagnostic creep’ is not limited to geography; diagnosis has also leaked outside of the clinic into the popular imagination.

With this popular fascination, we observe frequent diagnostic associations with celebrity or historical characters. Does Donald Trump have a narcissistic personality? Was Joan of Arc schizophrenic? What was the cause of Richard III’s hunchback? And even, does Winnie the Pooh have Obsessive Compulsive Disorder? Diagnoses are common currency for social commentary. While some of these extra-clinical diagnoses are completely speculative, and condemned by the APA, whose Code of Ethics specifically warns its members against diagnosing public figures whom they have never encountered professionally (McDaniel, 2016), they are nonetheless, commonplace.^1^

What is interesting in this fascination with the putative narcissism of Trump or even the psychic health of Jesus (Bundy, 1923) is less whether these diagnoses are valid and explanatory; and more, as Foxhall (2014) writes, ‘how, at the moment of creation, a diagnosis reflects the significance of particular medical signs and theories in historical context and how, when and why such diagnoses can come to do meaningful work when subsequently mobilised as scientific “fact”’. The process of diagnoses outside of the clinical setting is itself a historical artefact, a reflection of how we understand and categorise deviance in the late modern era, what Svend Brinkman refers to as our ‘diagnostic culture’ (Brinkmann, 2016). Brinkmann describes the shift in culture towards applying medical labels to oneself and others and interpreting troubles and differences in a diagnostic framework rather than in any other framework. Brinkmann highlights how our *diagnostic* frameworks are not the only way to interpret troubles, they have just become *dominant* ways in the here and now, and notably, dominant ways of telling stories.

This essay will explore a range of different kinds of non-clinical diagnoses, with a view to understanding the work they do. It will reveal how the range of pathographies capture, not the disorders of historical or fictitious figures, rather, the anxieties of a contemporary society, eager to explain deviance via diagnosis in order to make sense of the world. Rather than judging whether extra-clinic diagnosis is a worthwhile or ethical endeavour, we ask what function it performs.

We have chosen a broad sense of the word ‘pathography’ to describe these non-clinical diagnoses even though other uses have been made of the term. Dunglison (1853) introduced the term ‘pathography’ in his *Medical Lexicon in 1853, as* ‘a description of disease’ (p. 648). More recently its use is narrower. Some contemporary writers use the term to describe ‘historical biography from a medical, psychological and psychiatric viewpoint’ (Schioldann, 2003), what Muramoto (2014) has opted to call the ‘retrospective diagnosis’ (p. 2), or, to refer to stories of disease embedded in illness narratives by patients, about their diagnoses. Hawkins (1999), for example, uses the term pathography almost interchangeably with the term ‘illness narrative’ . We might be more inclined to think of what Hawkins refers to as ‘autopathography’, to story one’s own life in terms of illness, disorder or disease.

We have decided to stick with pathography in its etymological sense of the writing of disease (Gr. -Pathos – disease; graphos, to write or writer) and expand and differentiate as required as we explore the various forms of which there are several. We will include a range of different kinds of non-clinical diagnostic forms, from the autopathography (diagnostically-focussed account of self) to paleopathography (archaeological), psychopathography, and even fictopathography (a name we have coined to refer to the diagnosis of fictitious characters in popular culture).

In the pages which follow, we will review a number of these non-clinical diagnoses and offer an explanation for why they are so frequently used to ‘write’ disease. We will conclude with a discussion which illuminates the contemporary context Foxhall encourages us to interrogate.

## Pathographic forms: From paleopathography to fictopsychography

There are many kinds of extra-clinical diagnoses, from the analysis of archaeological remains to the illness narrative. They are differently motivated, but socially connected. We review some of these forms below.

### Paleopathography

Paleopathography is the analysis of archaeological remains in pursuit of diagnostic information. Its interest can be anchored in the intellectual curiosity of trying to understand the Way Things Were. Or its interest may be epidemiological. This history of a disease is something different than the history of an individual case. With the development of new genomic mapping techniques, some argue that we can construct epidemiologies of specific disorders to get a view using their ‘better’, modern understanding. For example, Alpha-1 antitrypsin (A1AT) deficiency is a hereditary disorder which is associated with significant risk of life-threatening lung and liver disease, but whose cause is poorly understood. The detection of its presence via genetic testing of archaeological sites, provides some clues. The study of frozen Inuit remains from 800 to 1200, of an exhumed 14th century Italian nobleman, and of a disinterred nineteenth-century Italian poet whose medical history was known all gave insights into ‘the natural history of pathological processes, whether due to genetic, infectious or environmental factors’ (Perciaccante et al., 2018: 3).

The analysis of remains may also serve to recover the identity of the corpse or learn more about the person whose remains are recovered. The recent case of King Richard III, which received extensive media attention, involved genetic material being extracted from remains at the former site of Greyfriars Abbey. This testing was both to confirm if the skeleton was that of the last of the last Plantagenet king, but also to find out the cause of his much-commented upon hunchback. By genetic and anatomical diagnosis of the skeletal remains, a new story of Richard III could be created. Not the malformed and limping humpback commemorated in Shakespeare’s play, where he is depicted as evil and deformed, we learn that his adolescent onset idiopathic scoliosis (Appleby et al., 2014), likely had no impact on his gait or general appearance (Mitchell, 2012, 2017). This case points to how historical representations are driven by the interests of their creators. Shakespeare may have been trying to curry favour with the Tudors, then in power at the time of his creative work. As with retrospective diagnoses of our era, Shakespeare’s assertions about Richard III are not only creative, they reflect a particular historical and political context. This is to say that there is a historical and political context for those who assert the scoliosis, or the ‘natural’ history of disease, as with A1AT.

Both these examples illustrate how people’s biographies are not only shaped by their newly minted pathographies, but how understandings of the pathologies themselves may also be altered in their retellings and in how stories are told. We can imagine, for example that A1AT now is thought of differently as a disease that has a genetic predisposition, but that its aetiology can incorporate the infectious and environmental discovered posthumously. In other words, the understanding of A1AT as a category has extended and been reshaped by the paleopathography. As paleopathographies, and other forms of pathography are incorporated into disease criteria, in a looping manner, standardised clinical diagnoses change conception of disease, and thus the pathographical inclusion criteria (Hacking, 1996).

### Psychopathography

Psychopathography is a much-debated form, where relics related to a specific historic individual are put in the public sphere to argue for a psychiatric disorder of some kind. The range of materials used for making this extra-clinical diagnosis are vast. These might include reports of the individual’s behaviours, notes from their medical file, commentaries by their peers, diaries, art works or writings by the individual. The case of Vincent Van Gogh is one with which most readers will be familiar. He died of a self-inflicted gunshot wound. Over 150 papers have been written to propose a range of diagnoses to explain his behaviours (Blumer, 2002). The various retrospective diagnoses have been established with reference to his contemporaneous diagnosis by doctor, Paul Gachet, his personal letters to his brother, his art work, his own description of symptoms and his consumption of absinthe (see e.g. Arnold, 2004; Coppola, 1950; Correa, 2014; Voskuil, 2013). Whether he had depression, bipolar disorder, or acute intermittent porphyria is a point upon which there is no agreement,^2^ but each diagnostic proposition is, in any case, an example of the desire to reconstruct an understanding of Van Gogh on the basis of a diagnosis.

Whether it is Van Gogh, Napoleon, Joan of Arc or Jesus, these psychopathographies, even when written by physicians, are not clinical diagnoses. They are derived from historical sources (in contradistinction to clinical signs). They serve the important role, however, of recounting the erratic, yet exceptional behaviours of the individuals concerned. Maybe to inspire, maybe simply to explain, these diagnoses make narrative sense of the exceptional, as they *write* the characters, and, to a certain degree, the diseases, via the diagnoses.

Muramoto’s strongly-argued critique of such diagnoses points out that not only are these historical sources being analysed and mobilised by clinicians hardly *au fait* with the discipline of historiography, frequently relying inappropriately on secondary or translated sources; they are interpreted without adequate understanding of, or engagement with, the context of the source (Muramoto, 2014). But further, he argues that what he calls the ‘retrospective diagnosis’ is ontologically flawed for a range of reasons: disease X can neither be considered nor experienced the same way in different eras. Similarly, disease X may be observer dependent. In any case ‘Retrospective diagnosis is anachronistic exactly because it tries to diagnose a disease of the past in contemporary terms’. (p. 4). To Muramoto, it only makes sense to apply the modern disease category in the modern setting. We do not agree. Retrospective diagnoses can be illuminating. They reframe history, and in so doing, highlight what is important in contemporary stories of difference and acceptable ways of being in today’s world.

Haridas (2015) points out that there is far less interest in diagnosing non-controversial characters, underlining our assertion above that diagnosis is used, even historically, to account for deviance. More attention has been directed to Tutankhamun’s ailments after his death that was ever likely in his lifetime; other prominent and controversial historical figures that have received the same pathographical attention, both their flaws and their exceptional determination redesignated as psychiatric include Pol Pot, Hitler, Marie Curie and Marilyn Monroe, (Kalb, 2016; Orrego and Quintana, 2007; Vachon, 2017). Whether it is controversy, and prominence, that directs the contemporary gaze towards diagnosis, the diagnosis provides an explanation for atypical behaviours, at the same time, revealing *how* at a given time, we explain the deviant, the uncommon.

More saliently, the extra-clinical diagnosis tells us little about the person diagnosed and much about the status and meaning of the diagnosis itself. The ever-increasing psychopathographic diagnoses of autism spectrum disorder provides a good example. The bestselling children’s book *Different Like Me: My Book of Autism Heroes* (Elder, 2005) lists Einstein, Warhol, Kandinsky, Turing, Tesla and Immanuel Kant as being ‘on the spectrum’. The website (‘History’s 30 Most Inspiring People on the Autism Spectrum’) claims both celebrities and historical figures including Charles Darwin, Stanley Kubrick, Michelangelo, Mozart and Sir Isaac Newton. This illustrates another way pathographies operate, to attach successful figures to a condition in order to reinforce self-worth and pride. These psychopathographies are frequently used to provide encouragement to autistic children, and to provide inspirational role-models. Autistic blogger, Forbes Wilcox (2011), describes Steve Jobs as autistic and a mercurial genius. He uses the diagnosis to explain both behaviours and talent, creating an association within his own group (autistic people) with the ‘genius’ Jobs. For activists, claiming Jobs as on-the-spectrum highlights autism as a condition to be proud of, one that confers huge value as well as challenges. Wilcox writes how Jobs and his kin ‘push the human race forward’. Thus, psychopathographies of prominent, rich famous and successful personalities help to reinforce affirmative view of conditions for some self-advocacy movements, whilst diagnosis of divisive figures (e.g. Donald Trump) are seen as detrimental the cause of other health advocacy groups.

In the case of both the living and the dead, psychopathography is used to describe extraordinary traits and enable an explanation for (what we consider) unusual behaviour that enables us to ‘come to terms’ with the actions. Historical review achieves thus, what Haridas refers to as a ‘hagiography’ or idealisation of the historical character. For Pol Pot or Hitler, retrospective diagnosis of a mental disorder allows us to come to terms with atrocity by explaining intent as an aberration of the mind.

Joan of Arc heard voices, dressed as a man and joined an army. She understood her experience of hearing voices as religious visions; a divine decree to diverge. She was eventually canonised in 1909. Yet, a divine explanation (the ‘master narrative’ of the time) holds little traction today, and in her psychopathography (Allen, 1975), the religious explanation is replaced by a diagnostic one. This case illustrates a disjuncture between explanations of mediaeval and modern eras, highlighting the ‘work’ of pathography. Clearly, we need to explain deviance. Be it Vincent Van Gogh, or Joan of Arc, in trying to make sense of how they engaged in socially deviant behaviours, we will turn to diagnosis. To explain assaults on gendered expectations, Marie Curie is today given the diagnosis of autism. It explains to the contemporary reader why she was so insensitive to family life and concentrated on scientific discovery, in an era which had other expectations for women. An exemption needs to be made in order to exculpate for non-conformance to gendered expectations of the day, or indeed of today. The diagnosis provides a narrative about this particular woman and her idiosyncrasies in a way that doesn’t disturb the popular belief about the gender order, in doing so both protecting and policing that order. Here diagnosis, the pathography has a political regulatory function.

### Diagnostic autopathography

We will use the term ‘autopathography’ to capture what Anne Hunsaker Hawkins described as ‘pathography’, the term we usurped, as described above, for its broader meaning.

Hawkins (1999) describes the pathography as ‘articulat[ing] the hopes, fears, and anxieties so common to sickness, but [also serving] as guidebooks to the medical experience itself, shaping a reader's expectations about the course of an illness and its treatment’ (p. 127). These accounts are what Frank (1993, 1998, 2016) refers to as ‘illness narratives’, and are how, he writes, people give meaning to their suffering. As the focus of this paper is on the performance of diagnosis outside of the clinic, we emphasise a distinct subset of autopathography, those focussed on diagnosis: either claiming one or demanding one in the absence of a diagnosis afforded in the clinical setting.

The demand for diagnosis is a theme of diagnostic autopathography which gives rise to creative, confessional, and advocacy texts. This is particularly salient in the relation to medically unexplained symptoms, or what Dumit (2006) calls ‘illnesses you have to fight to get’ (p. 577). The autopathography provides a potent weapon in this battle.

Yolanda Hadid’s *Believe Me: My battle with the Invisible Disability of Lyme Disease* is typical in this genre. This beautiful, successful TV star describes her descent into inexplicable illness, and her need to battle for recognition.

Another important form of diagnostic autopathography is the first-person illness documentary. These are films that are almost entirely focussed on self-representation, including archival footage such as family films/videos, photographs, written and/or visual diaries of the ill person, using a confessional style associated with an insistence on the ill person’s being in the world with their differences, yet, in pursuit of a diagnosis to legitimise their suffering. Without one, a person is left in a liminal state, betwixt and between, leaving them bereft of coherent ways to understand, to fix or to accept their situation. Pathographies are sometimes driven by the need for an explanation to fill the vacuum.

A film in this genre is Jennifer Brea’s *Unrest*. This film chronicles the filmmaker’s journey to make sense of her chronic fatigue, and her relentless pursuit of a diagnosis to explain it. This film describes a journey of rejection, self-doubt and marginalisation in medical and social contexts. The making of the film provides her with affirmation and agency as well as connection with a community of sufferers which whom she can connect: virtually and literally. She shifts roles from subject to interviewer and advocate deploying documentary strategies including participatory, performative, and self-reflexive approaches.

An interesting filmic variant of the autopathography is the fictionalisation of first-person account of illness, diagnosis and its consequences. *Pain and Glory*, directed by Pedro Almodóvar’s is drawn, as is all of his work, he says, from biographical experiences of sorts, but much altered for narrative purposes. His main character suffers from chronic back pain, addiction and medically unexplained symptoms.

These forms of autopathography are joined by VLOGS, BLOGS and other forms of social media which leverage the retelling of illness in pursuit of diagnosis. One example is Becca Halm’s ‘My Lyme Disease Story: Living with Chronic Illness’. This amateur youtube video features Becca Halm, leaning against her unmade bed telling a rambling story of chronic illness and associated symptoms which she attributes to the bite of a tick she asserts has infected her with Lyme disease, despite the implied protestations of the doctors who have been treating her (Halm, 2015). She describes how she was ‘finally allowed to be tested’ for Lyme disease, and uses her video as a call to arms, and plea for more research and awareness of this diagnosis.

Each of these media serve a different purpose and address a different audience, even though some (like *Unrest*) are boundary objects, occupying a space which spans different fields and intent, from cinematic to the activist, providing testimony, disease advocacy and documentary (among others). The medium is important because each one serves a different purpose and addresses different people. The documentary is a creative project which fit the particular conventions of that form whereas the blog may be creative but targets the community. But what binds them all is the public assertion ‘I have disease X/my mission to share my experience of disease X/to prove I have disease X’.

Rosenberg (2002) has written about the ‘tyranny of diagnosis’, a tyranny to which these personal diagnosis projects are committed. What is needed is to put a name to troubles, or experiences of disablement (Jutel, 2011). This can provide closure or a means by which to come to terms with dysfunction. What interpretation of troubles as pathologies may minimise in their translation as aspect of an individual’s disease or disorder is the political or social framework in which such troubles are often fostered.

### Fictopathography

We have coined the term fictopathography to refer to the diagnoses which are pronounced about characters who aren’t real. One such character is Johnny. He was character in the nineteenth century illustrated poems by German physician, Heinrich Hoffmann. The poem Johnny-head-in air (*Die Geschichte von Hans Guck-in-die-Luft*) published in 1876, goes like this:

As he trudged along to school,

It was always Johnny’s rule

To be looking at the sky

And the clouds that floated by;

But what just before him lay,

In his way,

Johnny never thought about;

So that everyone cried out

‘Look at little Johnny there,

Little Johnny Head-In-Air!’

This poem is credited with being the first reported ‘case’ of ADHD, albeit reported in a book of children’s poems, about a child who didn’t exist (Knight and Rappaport, 1999). While one might argue that ‘Little Johnny’ was distracted and hyperactive, one could just as easily argue, as have scholars of children’s literature, that this was a cautionary tale, written to curtail childhood excesses, and to entertain young children with the new genre of provocative and evocative illustrated picture books. ‘Children appreciated the drama and child-orientation of the stories as well as their anarchic spirit and grotesque exaggeration’; writes Metcalf (1996), ‘and in the case of Struwwelpeter (the book in which Die Geschichte von Hans Guck-in-die-Luft was published) parents presumably appreciated the ease with which children swallowed the nicely-wrapped educational message’(p. 201).

Characters in children’s books are prominent targets for fictopathography, with the tongue-in-cheek analysis of the characters in AA Milne’s *The House at Pooh Corner* (written, 1928), all diagnosed by Shea and colleagues ‘Pathology in the Hundred Acre Wood: A neurodevelopmental perspective on A.A. Milne’ (diagnosed, 2000), another example. Humorous, and intended as holiday reading, Shea et al’s paper was designed to entertain (Shea et al., 2000). But Pooh et al’s diagnoses have stuck. The story of Winnie-the-Pooh’s obsessive compulsive disorder, Roo’s autism, and Tigger’s ADHD, have been imported to scientific PowerPoint presentations (Humphrey, 2016) and teaching materials in schools. In these contexts, however amusing, the diagnoses illustrate the psychiatric categories as though they were on the mind of the author A.A. Milne. Pooh and his friends’ diagnoses are fluffy, non-threatening ways to introduce and talk about autism, ADHD and OCD to children. There seems to be a role these fictopathographies can play in contemporary culture as a way to deal with and manage societal anxieties about late modernity’s demand for docile students. Using a diagnosis of anxiety disorder, as for Piglet, not only positions the child as ill, but also deflects from the wider political debate about what situational factors lead to anxiety in the child population as a whole, what pressures and expectations children are under. The story shifts the site of intervention onto the child and away from the social and institutional norms that may have contributed.

Fictopathography is at work in diagnosis of cinema and television characters as well. The popular media is replete with psychiatric assessments of Arthur Fleck (the Joker) and his mother; with learned specialists picking up what the film ‘got wrong’, what diagnosis he might actually have, or what the impact of his putative diagnosis has on real-world sufferers of his candidate diagnoses. The number of diagnoses proposed for Joker include psychopathy (Tajjudin and Goodwin, 2019), Pseudobulbar affect (Pierce, 2019) or schizotypal personal disorder (Miller, 2019) and many more. Of course, what these analyses fail to emphasise is that Fleck doesn’t exist, and that the cinematic objective of suspending disbelief constructs a character not a patient. Director Todd Phillips, has commented that his creative intent was not for Joker to have a particular diagnosis, rather to show how the fraying of the social structure could lead to the creation of the Joker character (Gross, 2020).

Perhaps more powerfully than all the other forms, albeit with less consequence, the fictopathography underlines how important the diagnosis is to contemporary commentators for making sense of social behaviour. The possibilities are endless, as the foibles of any character can be recast as pathology. While many of the Joker comments were written to protest the presumption of Joker’s psychiatric illness – and the stigma it was presumed to impose more widely on people suffering from psychiatric illness – beyond his pseudobulbar affect (laughing at inappropriate moments) there was no mention of diagnosis in the film, other than that of his mother. And, it wasn’t his pseudobulbar affect that lead him to violence. Was he bad or was he sick? He was clearly deviant, and the rush to label him with a diagnosis is indeed in line with sociological explanations of diagnosis as the means by which deviance can be legitimised, or at least, understood (Conrad and Bergey, 2014; Conrad and Potter, 2000; Conrad and Schneider, 1992).

There are plentiful examples of fictopathography. Saga Noren is the Swedish detective, from the Scandinavian crime drama *The Bridge* who investigates homicides.^3^ Saga is just one fictional character of a panoply whose focussed behaviour has been attributed to autism, including Sheldon from *The Big Bang Theory*, Boo Radley in *To Kill a Mockingbird*, Forrest Gump of the titular film and Lisbeth Salander, *The Girl with the Dragon* tattoo (ABA Programs Guide, 2020). Saga’s diagnosis explains her peculiarities, her inability to engage in small talk, her disregard for social conventions (she often changes her clothes in public) and her obsessive fixation with her work, despite being beautiful and a successful investigator. The fictitious backstory blurs the explanation for Saga’s behaviour, suggesting an abusive mother, and her twin sister’s death by suicide. Saga’s popular diagnosis has been Asperger’s disorder (Townsend, 2015). The deliberate lack of clarity in the show whether Saga’s behaviours can be attributed to autism or perhaps to her traumatic past mirrors occasional dilemmas of clinicians in real world adult assessment settings, as they struggle to settle the diagnostic story (Hayes et al., 2020). Despite deliberate fuzziness of origin of her behaviour, Saga has been picked up and amplified as an icon of autistic women.

## Beyond humans

The extent of the practice of pathography is neatly illustrated by the instances of applying pathographies to pets. This is the anthropomorphising of pet diagnoses, where animal owners try to explain their animal’s behaviour via human diagnoses. Animal owners very likely draw on their own illness experiences not only when considering what ails their pets, but when conceptualising health and behaviour in general (Hobson-West and Jutel, 2020). A US based dog-care site for dog owners, *Wag!* provides the autism example:

*Can Dogs Get Autism?*

*YES!*

*In some dogs who are suffering from autism, repetitive behavior such as incessant tail chasing may be one of the more predominant symptoms. It is possible for the dog to become aggressive during an episode and care should be taken when approaching. In others, the condition may result in withdrawn behavior and a lack of activity. In some dogs, the symptoms may be so mild you* don’t *notice them, but if you suspect your dog may have autism, you take him or her to your veterinarian for diagnosis* (Wag!, 2019)

This process, the application of human diagnoses to companion animals, extends and legitimises the diagnostic category as much as it legitimises undesirable behaviour in pets. The category becomes universal, to the point where so firmly reified is a diagnosis it is now an idea that can be entirely separated and dislocated from the human body, and unproblematically transferred across the species boundary.

## Discussion

The contemporary social need to explain behaviour reveals how diagnosis has become a dominant explanatory device of our time for deviance in all its forms, with psychiatry guarding what Rosenberg (2006) refers to as the ‘ever-shifting boundary between disease and deviance’ (p. 407). But, it has also become a literary trope: a way of telling stories about transformation, about power and about deviance. As in the clinical setting, it reveals, in one word, a much larger collection of information.

Other diagnosers utilise pathography for specific arguments. Richard Dawkins effectively replaces religious visions with medical symptoms of hallucinations and delusions (*The God Delusion*, p. 90) as a counter argument to the existence of God (Dawkins, 2007). Using diagnosis, he discounts religious experience as an implausible narrative, invoking instead, ‘the truth’ of empirical science. The diagnostic narrative dovetails with his commitment to atheism.

Why do people make these diagnoses? There are a variety of reasons. Retrospective pathographies can reify modern diagnostic categories by giving the impression the modern disorder or disease, has always existed. And, they can be reified is across species boundaries. Diagnosis acts to construct a space in which the history and development of diseases as concepts are eradicated and they are replaced with a story where they have been true and present forever, and in all types of bodies (Wilson, 2013). Smith (2012) questions the idea of unchanging fixed categories in psychiatry showing how the rise of biological psychiatry led people to frame ADHD as a brain-based disorder. *Johnny-Head-In-*Air’s diagnosis of ADHD usurps a disciplinarian tale about a mis-behaving child, and transforming it into evidence of the presence of a neurological difference in ADHD academic and research literature (Banaschewski and Zuddas, 2018; Faraone et al., 2015).

The reification across time may be particularly important for disorders which are the poster children of medicalisation, like ADHD (Conrad and Bergey, 2014; Conrad and Potter, 2000; Conrad and Schneider, 1992). Demonstrating the universality and unchanging nature of behaviours that have prompted an ADHD diagnosis through time becomes imperative. The same legitimising function applies for those retroactively diagnosed with autism. According to Gernsbacher et al. (2005)

*..The phenomenon of autism has existed most likely since the origins of human society. In retrospect, numerous historical* figures. . .*fit autism diagnostic criteria but were not so diagnosed in their day (p. 55).*

The identification of autistic individuals through history helps confirm the biological, essential nature of the disorder. If people with ADHD and autism traits have existed in history, the categories can be seen as persistent and unchanging. Diagnoses thus come to do meaningful work when operationalised as scientific fact. As the diagnosis has been applied throughout history, it is universal and stable. The configuration of symptoms is sound and expression is not altered by historical context.

We can note the importance of persistent categories in essentialising phenomena via Hacking’s (2002) comment: ‘the idea of nature has served as a way to disguise ideology, to appear to be perfectly neutral. No study of classification can escape the obligation to examine the roots of this idea. . . no study of the word “natural” can fail to touch on that other great ideological word, “real”’ (Hacking, 2002: 7). Instead we see the promotion and differential allocation of resources to competing streams of knowledge in a ‘post-truth’ world, as what is ‘real’ becomes constructed.

We can return to the Winnie the Pooh characters’ diagnoses and see how they become knowledge objects with a life of their own. ‘Tigger has ADHD’ is a knowledge object, for example. His diagnosis now has its own life, repeated by generations of ADHD researchers to show ADHD has always been around. Students learn to relate this knowledge to real-life phenomenon when they encounter hyperactivity (Entwistle and Marton, 1994), and to use it as a heuristic for explaining ADHD. Tigger’s diagnosis becomes an active touchstone in creating knowledge about ADHD.

Not only do they explain, these diagnoses are at the same time, a call to action. Childhood diagnoses are now firmly a part of children’s landscape and language, consequently they should be addressed. Once a name is applied to a child’s behaviour, an intervention becomes *de rigueur.* The child, newly diagnosed with, be it anxiety, depression or attention deficit disorder, at the same time, becomes normalised and rebalanced by either medication or behavioural intervention. This subjectification of the child is mediated by the embrace of diagnostic language; stories about Winnie-the-Pooh provide non-threatening comparators on which to draw.

What cannot immediately be seen in the classroom, because we are currently in midst of the age of diagnosis, is that autism, ADHD, chronic fatigue, irritable bowel syndrome, Munchausen’s and the rest are unlikely to survive unchanged. Historians of the future may examine Tigger and Roo’s diagnoses as quirky artefacts illustrating what people thought about childhood behaviour ‘back in the old days’. Autism spectrum disorder again provides an excellent example of the mutability of diagnostic categories as demonstrated by the well-publicised sub-category of Asperger’s Disorder, once a diagnosis with ontological substance, now subsumed in the DSM-5 as part of a ‘new’ spectrum condition.

Pathography, as described in all its guises in the pages above, takes individuals, behaviours and indeed the broader world, and imposes an order which demands scrutiny from critical scholars.

The various pathographies show how diagnosis is an idea that can be removed from its context and transposed to dead people, fictional characters and animals. Although the classification of the various forms of pathography we present here stems more from ‘who’ is diagnosed rather than who is doing the diagnosing, the various practices of pathography reflect our contemporary understanding of disease and illness categories as the master narrative to explain deviance, and to legitimise the diagnoses themselves. In sum, the various pathology practices tell us more about us, the diagnosers, and their contemporary vision, than the lives of those they seek to describe.

The extra-clinical diagnosis serves as a reminder that we live in the age of diagnosis. The culture of storying and explaining lives by using diagnostic language shows the degree to which diagnosis is used in an explanatory capacity in high-income countries of the early twenty-first century. By defining dysfunctions as medical, we shut down other avenues of explanation, and, potentially, risk losing touch with the wider context in which such pathological frameworks operate (Brinkmann, 2016). In this sense, pathographies are a form of medicalisation because they simultaneously expand the signifiers of illness and disorders, and extend the diagnostic language we have to talk about them, reducing to individuals’ bodies and brains what may have been politically or contextually driven. The various pathographies we have identified here reflect current diagnostic and popular culture as well as create the semiotics that mediate contemporaneous understandings of people’s experiences. This is an understanding that can be useful, but attention on the various pathographic forms highlight overdue consideration of their own place in the bigger political and historical picture. Understanding diagnosis outside the clinic helps to highlight the extension of diagnosis and its place in understanding health and disease. Through pathography, diagnosis creeps into everyday domains it has not occupied previously, a form of medicalisation through popularisation. Pathography emphasises the importance of diagnosis as a social agreement rather than a label for an immutable fact of nature.

## Notes

1. This warning is often referred to as the Goldwater Rule, in reference to the case of a 1964 candidate to the US presidency, Barry Goldwater, who was portrayed as emotionally unstable, delusional and paranoid in an article citing a dubious poll of American psychiatrists (Lilienfeld et al., 2018), and probably contributing to his defeat in the subsequent election.

2. There are over 30 different diagnoses proposed

3. She is renamed Sonya Cross in the US remake.
